# Exploring the Relationship Between Self-Employment and Health Among Blacks

**DOI:** 10.1089/heq.2019.0084

**Published:** 2020-01-23

**Authors:** Kimberly Danae Cauley Narain, Kia Skrine Jeffers

**Affiliations:** ^1^ Division of General Internal Medicine and Health Services Research, Department of Medicine, David Geffen School of Medicine and Center for Health Advancement, University of California, Los Angeles, Los Angeles, California.; ^2^ School of Nursing, University of California, Los Angeles, Los Angeles, California.

**Keywords:** self-employment, health disparities, hypertension, exercise, diet

## Abstract

**Purpose:** There is some evidence that self-employment may improve measures of cardiovascular and general health among the general population; however, no studies have examined this relationship among Non-Hispanic Blacks (NHBs). Studying the health implications of self-employment among NHBs is important because of the disparities that persist in both cardiovascular health and self-employment rates between NHBs and other racial/ethnic subgroups.

**Methods:** A pooled cross-sectional analysis of data from the Behavioral Risk Factor Surveillance System (2000 to 2014) was used to explore the association between self-employment and the following self-reported outcomes: “no exercise,” fruit consumption, vegetable consumption, days of alcohol consumption, fair or poor health, hypertension, poor mental health days, and poor physical health days among the total population of NHBs and across gender/income subgroups.

**Results:** We find favorable associations between self-employment and several measures of cardiovascular health (increased fruit and vegetable consumption, reduced reports of “no exercise,” and reduced reports of hypertension) and positive associations between self-employment, poor mental health days, and days of alcohol consumption among the total population. The nature of these associations varies across gender/income subgroup.

**Conclusions:** Given the disparities between racial/ethnic subgroups with respect to adverse cardiovascular outcomes and the well-documented roles of exercise and blood pressure control in limiting cardiovascular disease, it is important to probe the relationship between self-employment and health among NHBs further.

## Introduction

People who are Non-Hispanic black (NHB) suffer disproportionate morbidity and mortality from cardiovascular disease (CVD), relative to people who are Non-Hispanic white (NHW). For example, NHBs have a stroke risk more than double that of NHWs and they are 30% more likely to die from heart disease. Additionally, the age of onset of CVD is younger among NHBs, relative to NHWs.^1^ A key driver of CVD disparities among NHBs is suboptimal cardiovascular health measures. Brown et al. found that the percentage of NHBs with optimal Life's Simple 7 scores (a measure that includes blood pressure, cholesterol, hemoglobin A_1c_, body mass index, physical activity, diet, and smoking) was 22 and 8 percentage points less, relative to NHWs, ages 25–44 and ≥65, respectively.^2^

There is evidence that self-employment is associated with improved cardiovascular health measures among the general population. For instance, Yoon et al. found that self-employment was positively associated with physical exercise and normal weight when controlling for demographics, education, income, family structure, access to health care, occupation type, and census region. The researchers also found negative associations between self-employment and diabetes, hypertension and high cholesterol in adjusted models.^3^

Other studies have shown that self-employment may have different health effects if it is in response to exclusion from the traditional labor market (necessity entrepreneurship) instead of in response to a business opportunity (opportunity entrepreneurship), with the positive health benefits of self-employment accruing to opportunity entrepreneurs.^4^ Studying the health implications of self-employment among NHBs is important because of the disparities that persist in both cardiovascular health and self-employment rates between NHBs and other racial/ethnic subgroups. A report from the Pew Research Center lists self-employment rates at 11%, 10%, 8%, and 5% for NHWs, Asians, Hispanics, and NHBs, respectively.^5^

The independence that the self-employed possess may buffer the effects of high workloads and job strain, potentially reducing work-related stress and improving health outcomes.^6^ Increased work flexibility may allow more time for engagement in health promoting behaviors such as exercise.^3^

Among NHBs, self-employment may provide an additional buffer against actual or perceived workplace discrimination. Work-place discrimination has been linked with stress, worse self-reported health, hypertension, and higher allostatic load among NHBs.^7–11^ The protective effect of education against occupational stress has also been found to be lower among NHBs relative to NHWs.^12^ Consequently, work-related stress generally and perceived workplace discrimination in particular, may play a role in the CVD morbidity and mortality disparities observed among NHBs. Conversely, self-employed NHBs may face more financial uncertainty, relative to NHB wage workers, potentially increasing stress levels and worsening health outcomes.^13^

To our knowledge, no studies have explored the relationship between self-employment and health among NHBs. We pool several years of Behavioral Risk Factor Surveillance System (BRFSS) survey data and use a cross-sectional study design to examine the association between self-employment and several different measures of self-reported health including measures of cardiovascular, mental, and physical health.

## Methods

### Study design and patient population

This study analyzes data from the BRFSS, covering the 15-year period from 2000 to 2014. The BRFSS is conducted by state health departments, with technical and methodological assistance provided by the Centers for Disease Control and Prevention. This study sample includes individuals between 21 and 64 who self-identified as NHB and reported their current employment status.

### Self-employment measure

Our primary predictor, “self-employment,” is a dichotomous variable based off responses to the question on current employment status. Self-employment was coded as “1” if the response was “self-employed” and coded as “0” if the response was “employed for wages.”

### Health outcome & health behavior measures

Four health behavior outcomes were examined: (1) exercise; (2) alcohol consumption; (3) fruit intake; and (4) vegetable intake. An indicator for “no exercise in the last 30 days” was coded as “1” if the condition was present. Health behavior variables for fruit and vegetable intake were count variables based on consumption during the last 30 days. A measure of the number of days alcohol was consumed in the past 30 days was also a count variable. We examine the days alcohol was consumed in the last 30 days because there is evidence that even moderate daily drinking may increase the risk of developing liver disease.^14^

Four self-reported health outcomes were examined: (1) fair or poor health; (2) diagnosis of hypertension; (3) poor mental health days; and (4) poor physical health days. Self-reported fair or poor health and self-reported hypertension were indicator variables coded as “1” if the condition was present.^9^ Self-reported poor mental health days and poor physical health days were treated as count variables based on the last 30 days.

### Other measures

Several individual-level variables that may be related to both self-employment and self-reported health outcomes were included in the adjusted models (age, gender, marital status, education, annual household income tercile (low <25 K, middle [25 to <50 K], and high [≥50 K]), presence of minor children in the home, health insurance coverage, and having a primary care provider) (Table 1). We also included a measure of 1-year lagged state gross domestic product (GDP) to capture state-level economic fluctuations. Finally, we included state and year-level fixed effects in the model to control for any time-invariant state characteristics and national policies, which may have impacted both self-employment decisions and health outcomes.

**Table 1.** Descriptive Statistics

	Mean (SD) or %
Health behaviors	
No exercise in the last 30 days	26.6
^1^Days ETOH consumed in the last 30 days	4.4 (6.6)
Times fruit consumed in the last 30 days	24.7 (28.9)
Vegetable servings consumption in the last 30 days	27.5 (26.8)
Health outcomes	
Self-reported hypertension	34.3
Self-reported fair or poor health	11.5
Poor mental health days in the last 30 days	3.2 (7.2)
Poor physical health days in the last 30 days	2.2 (5.8)
Covariates	
No high school	7.6
High school diploma/GED	63.4
College or higher	29.1
Age	39.9 (10.2)
Female	52.5
Married	43.1
Minor children	53.6
Low income	29.7
Middle income	35.7
High income	34.7
No health insurance coverage	19.7
No primary medical care provider	23.1
1-year lag GDP %	3.84 (2.7)

The data source is BRFSS (2000–2014 panels).

BRFSS, Behavioral Risk Factor Surveillance System; ETOH, alcohol; GDP, gross domestic product; GED, general education development; SD, standard deviation.

### Analysis

We calculated means and proportions for continuous/count variables and dichotomous variables, respectively (Table 1). We compared means and proportions across employment status using *t*-test and test of proportions, respectively (Table 2). For adjusted analyses, Logistic Regression Models (LRM) and Poisson Regression Models (PRMs) were estimated for dichotomous and count outcomes (Table 3). We used a PRM to model count outcomes instead of an Ordinary Least Squared regression model because the normality assumption may not be applied to count data even in the context of large sample sizes if the number of counts is small. Additionally, use of the PRM will account for the nonlinearity of count data.^10^ All models were weighted to account for complex survey design and nonresponse by using BRFSS sampling weights. Additionally, robust standard errors clustered at the state-level were used. We employed these adjustments through the Stata Survey command with the linearized option in Stata version 13 (StataCorp LP, College Station, TX). The results of the LRMs and PRMs are presented as odds ratios (ORs) and rate ratios (RRs), respectively. The level of statistical significance was *p* value ≤0.05.

**Table 2.** Bivariate Analyses for Patient Characteristics Across Employment Status

Patient characteristic or experience mean (SD) or ***n*** (%)	Self-employed	Employed for wages	***p***
Health behaviors			
No exercise in the last 30 days	29	25	0.00
^1^Days ETOH consumed in the last 30 days	5 (7)	4 (6)	0.00
Times fruit consumed in the last 30 days	26 (29)	24 (28)	0.00
Vegetable servings consumption in the last 30 days	34 (29)	33 (29)	0.00
Health outcomes			
Self-reported hypertension	31	35	0.00
Self-reported fair or poor health	14	12	0.00
Poor mental health days in the last 30 days	3.2 (7)	3.1 (7)	0.02
Poor physical health days in the last 30 days	2.5 (6)	2.3 (6)	0.00
Covariates			
No high school	10	7	0.00
High school diploma/GED	59	62	0.00
College or higher	30	31	0.00
Age	44 (11)	43 (11)	0.00
Female	50	67	0.00
Married	42	37	0.00
Minor children	47	50	0.00
Low income	33	27	0.00
Middle income	27	33	0.00
High income	30	32	0.00
No health insurance coverage	39	16	0.00
No primary medical care provider	71	82	0.00
1-year lag GDP %	4 (3)	4 (3)	0.67

The data source is BRFSS (2000–2014 panels). Bivariates for dichotomous and count/continuous variables are calculated using the test of proportions and *t*-test, respectively.

**Table 3.** The Relationship of Self-Employment to Health Behaviors and Health Outcomes Among Blacks

	Health behaviors				Health outcomes			
	No exercise (OR)	Fruit consumption (RR)	Vegetable consumption (RR)	Alcohol consumption (RR)	Self-reported fair or poor health (OR)	Self-reported HTN diagnosis (OR)	Poor mental health days (RR)	Poor physical health days (RR)
All genders	0.73 (0.67, 0.78) *n*=179,270	1.10 (1.04, 1.16) *n*=87,945	1.07 (1.03, 1.11) *n*=87,474	1.16 (1.10, 1.22) *n*=129,712	1.02 (0.93, 1.13) *n*=181,315	0.79 (0.71, 0.87) *n*=96,726	1.11 (1.03, 1.19) *n*=175,650	0.98 (0.91, 1.06) *n*=175,157
Men								
Low	0.86 (0.72, 1.03) *n*=12,863	1.09 (0.96, 1.25) *n*=6185	1.08 (0.96, 1.22) *n*=6114	1.09 (0.98, 1.21) *n*=9971	1.23 (0.99, 1.51) *n*=13,099	0.76 (0.59, 0.98) *n*=7090	1.10 (0.91, 1.32) *n*=12,639	1.10 (0.92, 1.32) *n*=12,576
Middle	0.75 (0.61, 0.92) *n*=19,317	1.08 (0.91, 1.28) *n*=9442	1.02 (0.92, 1.13) *n*=9377	1.02 (0.90, 1.15) *n*=14,909	0.93 (0.72, 1.18) *n*=19,507	0.68 (0.51, 0.91) *n*=10,749	1.12 (0.92, 1.37) *n*=18,817	0.70 (0.56, 0.86) *n*=18,763
High	0.92 (0.74, 1.14) *n*=23,837	1.15 (1.02, 1.30) *n*=11,736	1.12 (1.03, 1.23) *n*=11,667	1.06 (0.96, 1.17) *n*=19,045	1.21 (0.89, 1.65) *n*=24,090	0.80 (0.64, 1.00) *n*=12,453	1.29 (1.03, 1.61) *n*=23,495	0.88 (0.71, 1.10) *n*=23,494
Women								
Low	0.68 (0.58, 0.79) *n*=35,703	1.19 (1.07, 1.33) *n*=17,478	1.17 (1.07, 1.27) *n*=17,365	1.17 (1.01, 1.36) *n*=24,205	0.96 (0.80, 1.16) *n*=36,084	1.07 (0.85, 1.36) *n*=19,518	1.15 (1.02, 1.30) *n*=34,828	1.12 (0.96, 1.32) *n*=34,632
Middle	0.67 (0.56, 0.81) *n*=38,803	1.15 (1.02, 1.30) *n*=19,281	1.10 (1.02, 1.19) *n*=19,215	1.03 (0.87, 1.20) *n*=26,682	0.82 (0.65, 1.04) *n*=39,156	1.02 (0.79, 1.31) *n*=21,247	0.93 (0.80, 1.08) *n*=37,908	0.94 (0.76, 1.16 *n*=37,801
High	0.78 (0.62, 0.98) *n*=35,362	1.12 (0.99, 1.26) *n*=17,711	1.06 (0.98, 1.14) *n*=17,628	1.30 (1.13, 1.49) *n*=25,944	0.72 (0.54, 0.97) *n*=35,622	0.60 (0.46, 0.79) *n*=18,389	0.98 (0.82, 1.17) *n*=34,879	0.80 (0.66, 0.96) *n*=34,871

The data source is BRFSS (2000–2014 panels). Logistic Regression (LR) and PRMs are used to examine dichotomous and count outcomes, respectively. All models control for age, gender, marital status, education, presence of minor children in the home, health insurance coverage, having a primary care provider, 1-year lagged GDP, and year and state fixed effects. All models are weighted for complex survey design and nonresponse. Total population models also control for gender and income. Standard errors are robust and clustered at the state level. Results of LRs and PRMs are presented as ORs and RRs, respectively.

ORs, odds ratios; PRMs, Poisson Regression Models; RRs, rate ratios.

Given the potential for differential associations between self-employment and health outcomes, contingent on entrepreneurship type (necessity vs. opportunity), we evaluate the effects of self-employment across income tercile, in effort to illuminate relationship heterogeneity in these different employment contexts.^15^ We use household income as a proxy measure for entrepreneurship type because studies have consistently shown higher levels of household income among opportunity entrepreneurs, relative to necessity entrepreneurs.^16^

Women, both working for wages and self-employed, are typically in different job sectors relative to men, which may have different implications for associated health behaviors and health outcomes.^17,18^ NHB self-employed women are clustered in service businesses such as hair salons, catering, child daycare centers, and consulting. There is also research showing that the health impacts of social determinants such as income and employment may vary across gender and race.^19–21^ To assess for interactions between entrepreneurship type, gender, and self-employment we also run models stratified by household income and gender.

## Results

In bivariate analyses, relative to wage workers, the self-employed were older, more likely to be male, less likely to have a high school diploma/general educational development or college degree, more likely to report low-income, more likely to be married, less likely to have minor children, and less likely to be insured or have a primary care provider. The self-employed were also less likely to report “no exercise” and a diagnosis of hypertension, but were more likely to report fair or poor health. Lastly, the self-employed reported higher levels of fruit and vegetable intake and alcohol consumption, and more poor mental health days (Table 2).

The results for the adjusted analyses are found in Table 3. The results for the total population are listed in the first row. With respect to health behaviors, self-employment was negatively associated with reporting “no exercise” (OR=0.77; 95% CI=0.72–0.83) and positively associated with fruit intake (RR=1.12; 95% CI=1.07–1.19), vegetable intake (RR=1.09; 95% CI=1.04–1.13), and alcohol consumption (RR=1.08; 95% CI=1.03–1.14), controlling for other factors.

These findings translate into a 13% reduction in the odds of reporting “no exercise” and a 12% increase in fruit intake (roughly 3 more pieces of fruit per month), a 9% increase in vegetable intake (2.5 more servings of vegetables per month), and an 8% increase in alcohol intake (alcohol consumed on 0.4 more days per month) among the self-employed relative to wage workers. Self-employment was negatively associated with reports of hypertension (OR=0.79; 95% CI=0.71–0.87) and positively associated with an increase in poor mental health days (RR=1.11; 95% CI=1.03–1.19). These findings translate into a 21% reduction in the odds of reporting hypertension and an 11% increase in poor mental health days (an increase of 0.4 days of poor mental days per month) among the self-employed, relative to wage workers.

### Association of self-employment with health and health behaviors among men

Among men in the low-income subgroup; self-employment is associated with reduced reports of hypertension (OR=0.76; 95% CI=0.59–0.98). None of the other outcomes are significantly associated with self-employment in this population. Among men in the middle-income subgroup, self-employment is negatively associated with reports of no exercise (OR=0.75; 95% CI=0.61–0.92), hypertension (OR=0.68; 95% CI=0.51–0.91), and poor physical health days (RR=0.70; 95% CI=0.56–0.86). Among men in the high-income group self-employment is positively associated with fruit (RR=1.15; 95% CI=1.02–1.30) and vegetable intake (RR=1.12; 95% CI=1.03–1.23) and reported poor mental health days (RR=1.29; 95% CI=1.03–1.61).

### Association of self-employment with health and health behaviors among women

Among women in the low-income subgroup, self-employment is negatively associated with reports of “no exercise” (OR=0.68; 95% CI=0.58–0.79) but positively associated with fruit intake (RR=1.19; 95% CI=1.07–1.33), vegetable intake (RR=1.17; 95% CI=1.07–1.27) and poor mental health days (RR=1.15; 95% CI=1.02–1.30). Among women in the middle-income group self-employment is also negatively associated with reports of “no exercise” (OR=0.67; 95% CI=0.56–0.81) and positively associated with fruit intake (RR=1.15; 95% CI=1.02–1.30) and vegetable (RR=1.10; 95% CI=1.02–1.19) intake. However, there is no association between self-employment and poor mental health days in this subgroup. Among women in the high-income subgroup, self-employment is positively associated with days of alcohol consumption (RR=1.30; 95% CI=1.13–1.49), but negatively associated with reports of “no exercise” (OR=0.78; 95% CI=0.62–0.98), fair or poor health (OR=0.72; 95% CI=0.54–0.97), hypertension (OR=0.60; 95% CI=0.46–0.79), and poor physical health days (RR=0.80; 95% CI=0.66–0.96).

## Conclusion/Discussion

There is evidence linking self-employment with improved measures of cardiovascular and general health. Increased workplace autonomy and flexibility are proposed to underlie these relationships. On average, NHBs have lower levels of work-place decisional-authority and report relatively high levels of work-place discrimination, both of which are negatively associated with measures of cardiovascular health.^8,9^ To start to investigate the relationship between self-employment, cardiovascular health, and health outcomes generally among NHBs, we conducted a pooled cross-sectional analysis of 15 years of BRFSS data.

Among the total population, our findings show favorable associations between self-employment and several measures of cardiovascular health (increased fruit and vegetable consumption, reduced reports of “no exercise,” and reduced reports of hypertension). However, there are also positive associations between self-employment, poor mental health days and days of alcohol consumption. In gender/income stratified analyses we find that the associations of self-employment with improved dietary quality are only significant among women in the low and middle-income groups and among men in the high-income group. The negative association between self-employment and reports of “no exercise” is observed among all subgroups of women, but only among the middle-income subgroup of men.

We find a negative association between self-employment and reported hypertension only among the high-income subgroup of women and among all subgroups of men, except the high-income subgroup. The positive association between self-employment and poor mental health is only observed among women and men in the low and high-income groups, respectively. The positive association between self-employment and days of alcohol consumed is observed among the low and high-income subgroups of women. Among men and women in the high and middle-income subgroups, respectively, self-employment is negatively associated with poor physical health days. Among women in the high-income subgroup self-employment is also negatively associated with reports of fair or poor health.

These findings must be interpreted in the context of several limitations. Given that this study is cross-sectional, reverse causality cannot be ruled out as an explanation for these findings. Individuals with chronic diseases, such as hypertension, may be less likely to choose self-employment due to the desire to maintain employer-sponsored health insurance benefits.^22^ There is also evidence that individuals with mental illness may be drawn to self-employment.^23^

Additionally, there may be characteristics that simultaneously impact the propensity to choose self-employment and health outcomes.^24^ We also cannot guarantee that individuals who report self-employment are not also engaged in work for wages. This is particularly relevant for NHB women who often start businesses on the side while maintaining work for wages.^18^ Additionally, we have no way of knowing how long someone has been self-employed and no means of controlling for rural or urban location designation. We also use self-reported health measures, which have been shown to be a more reliable and valid predictor of health outcomes in NHWs relative to racial/ethnic minorities, among individuals with routine access to health care and among individuals with higher socioeconomic status.^25^

We take these validity limitations into consideration by controlling for health insurance coverage and having a primary care provider. We also consider several other factors that can be sources of omitted variable bias in our analyses such as education, household income, family structure, differences in state-level characteristics and year-specific trends. Additionally, we attempt to assess for differential associations between self-employment and health outcomes across entrepreneurship type, although using the somewhat endogenous measure of household income. Lastly, since our models adjust for several socioeconomic status factors that are correlated such as household income and education our results may be subject to collider-stratification bias.^26^

Despite the study limitations, there are some important findings such as a relatively large negative association between self-employment and reports of “no exercise” among all subgroups of women and middle-income men. Additionally, this study finds a large negative association between self-employment and reported hypertension among several gender/income subgroups. Given the disparities between racial/ethnic subgroups with respect to adverse cardiovascular outcomes and the well-documented roles of exercise and blood pressure control in limiting CVD, it is important to probe the association between self-employment and health among NHBs further, using study design capable of identifying causal relationships.^27^

The negative association between self-employment and reports of hypertension only being observed among the high-income subgroup of women may be consistent with the results of other studies that have found that the health benefits of entrepreneurship only extend to opportunity entrepreneurs.^4^ Many of the self-employed women in the low and medium-income subgroups may be necessity entrepreneurs. According to a 2018 report commissioned by American Express on the state of women-owned business, NHB women are often necessity entrepreneurs. In the necessity entrepreneur context, stress from financial concerns may limit health benefits associated with increased work autonomy and flexibility.

A high proportion of necessity entrepreneurship among the low-income subgroup of women might also explain the positive relationship between self-employment and poor mental health days observed in this subgroup. The finding of a positive relationship between self-employment and poor mental health days among high-income men is inconsistent with the idea of necessity versus opportunity entrepreneurship. These findings may reflect a stressful business context experienced by this subgroup of men that competes with the psychological benefits of work autonomy and flexibility. NHB men in the high-income group may be operating businesses in high-entry barrier sectors such as finance, insurance, and business services.

On average these businesses are relatively new and the closure rates are high.^28^ Alternatively, these findings may be the results of reverse causality given that men with mental illness may be drawn to self-employment in high-entry barrier sectors.^23^ There is also evidence that suggest that having a high household income is actually a risk factor for depression among NHB men when other socioeconomic status factors are taken into account.^20^

Future studies should use longitudinal data, more valid health measures, and more methodologically robust study designs to examine the causal nature of the relationship between self-employment and health among NHBs. If subsequent studies find the positive relationship between self-employment, dietary quality, exercise, and improved blood pressure control observed for some subgroups to be causal self-employment may be one strategy for buffering some of the deleterious health effects of systemic racism. Consequently, more emphasis can be placed on the development of interventions to promote self-employment among NHBs.

Specifically, lower levels of education, limited assets, lack of role models and discriminatory lending practices constrain self-employment opportunities among NHBs and each of these factors may serve as potential intervention targets.^28^ Additionally, studying the characteristics of the self-employed individuals within these subpopulations may provide insight into potential intervention targets for improving dietary quality, exercise, and blood pressure control in the broader population of NHBs. If the relationship between self-employment and poor mental health outcomes observed among women and men in the low and high-income subgroups, respectively, proves to be causal in subsequent studies it will be important to develop interventions to better support the mental health of individuals pursuing self-employment in these subpopulations.

## Abbreviations Used

BRFSS: Behavioral Risk Factor Surveillance System
CVD: cardiovascular disease
ETOH: ethanol
GDP: gross domestic product
LR: Logistic Regression
LRM: Logistic Regression Models
NHBs: non-Hispanic Blacks
NHW: non-Hispanic white
OR: odds ratio
PRMs: Poisson Regression Models
RR: rate ratio
SD: standard deviation
SES: socioeconomic status
